# Congenital Cerebral Palsy, Child Sex and Parent Cardiovascular Risk

**DOI:** 10.1371/journal.pone.0079071

**Published:** 2013-11-01

**Authors:** Elani Streja, Chunsen Wu, Peter Uldall, Jakob Grove, Onyebuchi Arah, Jørn Olsen

**Affiliations:** 1 Department of Epidemiology, School of Public Health, University of California Los Angeles, Los Angeles, California, United States of America; 2 Section for Epidemiology, Department of Public Health, Aarhus University, Aarhus, Denmark; 3 The Danish Cerebral Registry, National Institute of Public Health, Southern University, Odense, Denmark; 4 Department of Pediatric, Rigshospitalet, University of Copenhagen, Copenhagen, Denmark; 5 Institute of Biomedicine and Bioinformatics Research Centre, University of Aarhus, Aarhus, Denmark; Université de Montréal, Canada

## Abstract

**Objective:**

Genes associated with cardiovascular disease may also be risk factors for congenital cerebral palsy (CP) and these associations may be modified by sex, since there is an increased risk of CP in male children. We investigated the association between CP of the child with cardiovascular disease in parents, taking sex of the child into consideration.

**Methods:**

All parents of non-adopted singletons born in Denmark between 1973 and 2003 were included. Parents of a child with CP, confirmed by the Danish National CP registry, were considered exposed. Cox proportional hazards regressions were used to model risk of cardiovascular outcomes for exposed parents compared to all other parents beginning at the child’s 10^th^ birthday.

**Results:**

We identified 733,730 mothers and 666,652 fathers among whom 1,592 and 1,484, respectively, had a child with CP. The mean age for mothers at end of follow up was 50±8 years. After adjustment for maternal age, parental education, child’s sex, child’s residence, child being small for gestational age and maternal hypertensive disorder during pregnancy, mothers of CP male children had an excess risk of cardiovascular disease (HR: 1.52, 95% CI: 1.16-2.00), attributable mostly to an increased incidence of hypertension and cerebrovascular disease. After additional adjustment for preterm birth, the association was markedly attenuated for cardiovascular disease (1.34, 95%CI: 1.02 - 1.76), became nonsignificant for hypertension, but remained significant for cerebrovascular disease (HR: 2.73, 95% CI: 1.45- 5.12). There was no increased risk of cardiovascular events in mothers of female CP children, or fathers of CP children of any sex.

**Conclusions:**

Women that have a male child with CP are at increased risk for premature cardiovascular disease. Part of this association may be related to risk factors for preterm births.

## Introduction

Pregnancy related conditions have been identified as important risk factors for cardiovascular diseases in women. Recently, the *Effectiveness-Based Guidelines for the Prevention of Cardiovascular Disease in Women* recommend that “healthcare professionals who meet women for the first time should take a careful and detailed history of pregnancy complications with focused questions on the history of gestational diabetes mellitus, preeclampsia, preterm birth, or birth of an infant small for gestational age”[1], all risk factors for cardiovascular disease. 

Congenital cerebral palsy (CP) is the most common severe physical developmental disorder in children occurring with a birth prevalence of 2 per 1000 live births [2,3]. Hypertensive disorders of pregnancy (preeclampsia and/or gestational hypertension), preterm birth, or birth of an infant small for gestational age are major risk factors for CP and account for more than 50% of the cases[4]. It is not known if delivery of a child with CP is also a marker for increased cardiovascular risk in the parents. Should CP be associated with increased risk of cardiovascular disease in the parents, it would be important to know if this risk is independent from the above listed other risk factors for CP and cardiovascular disease. In addition, women carrying a male fetus are at higher risk of preterm delivery [5]. Consequently, it is important to investigate if child sex plays a role in the association between having a child with CP and parent’s development of cardiovascular outcomes.

We hypothesized that parents who have a child with CP are at an increased risk for cardiovascular outcomes earlier in life compared to parents who do not have a child with CP. We also examined if this potential association may be modified by the sex of the child.

## Methods

### Study Population

Denmark has one of the world’s most comprehensive registration systems with extensive population based data on health and social conditions [6]. All residents in Denmark are assigned a unique personal identification number at birth, which enables accurate linkage of individual information among all Danish national registries. Notification of a child’s birth is mandatory in Denmark, and records provide identification of the child’s biological parents, which enables identification of families in two or more generations. The Danish Medical Birth Registry, established in 1968 and computerized in 1973, contains medical data on all live and still births in Denmark[7]. 

**Figure 1:**
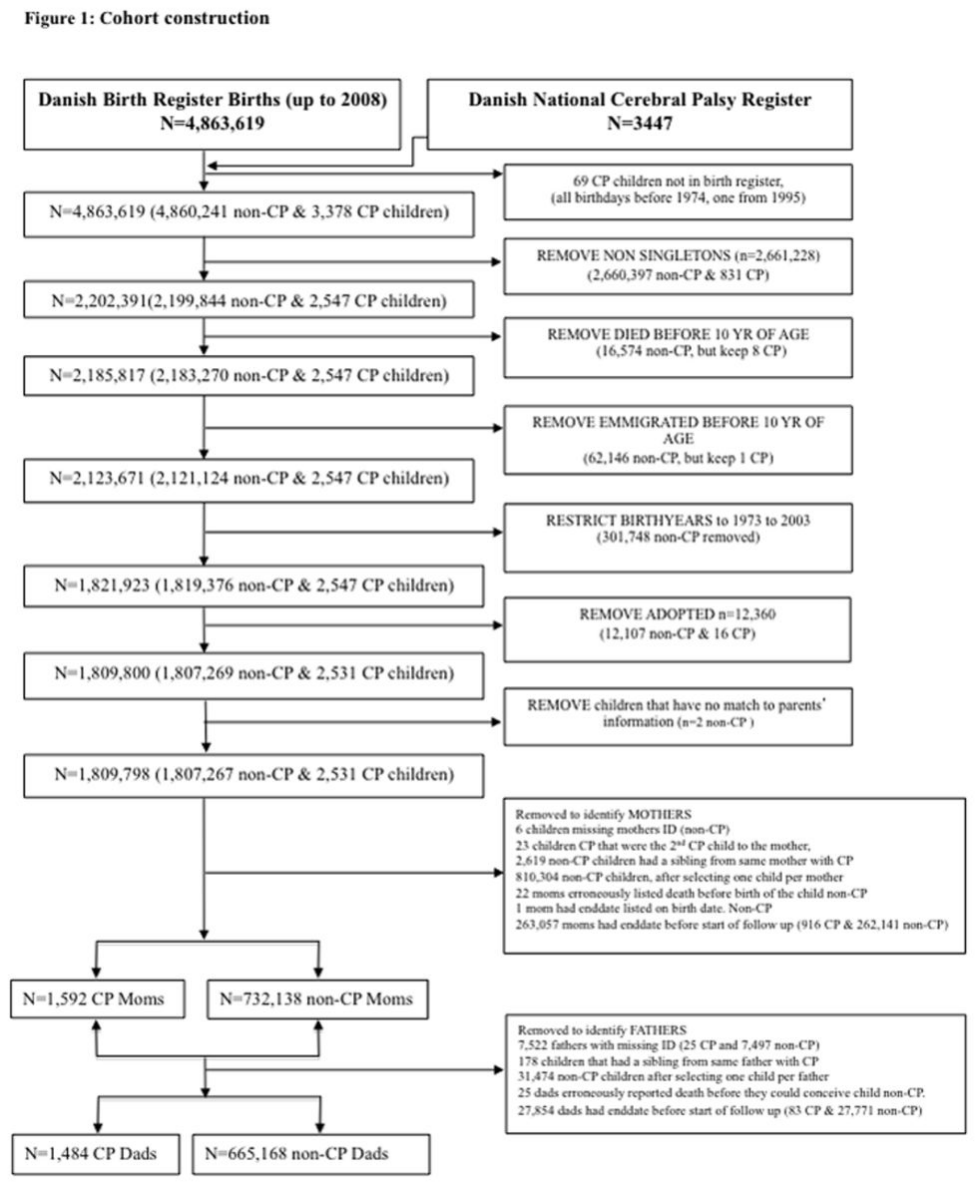
Algorithm of study cohort creation.

We identified all live-born non-adopted singletons born in Denmark between January 1, 1973 and December 31, 2003 (see Figure 1). Of these, 1,809,798 were alive and did not emigrate from Denmark before age 10 years and were linked to their parents’ records. We first identified mothers of CP children. If the mother had more than one child with CP (n=23), we randomly selected either the first or second child to be used for the study. We then identified mothers without any children with CP and randomly selected one child per mother to be used as the index child, adjusting for maternal parity. From the mother’s cohort, we identified the child’s father and removed all children where fathers could not be identified (n= 7,522). We similarly identified one father per CP child and removed his or her non-CP sibling. Then for remaining non-CP children, we randomly selected one non-CP child per father to be used as the index child. We additionally removed any parents with coding errors, such as a date of death prior to a time period where they could have conceived the child (day of birth of child -315 days), (n=22 mothers and n= 25 fathers). We began follow up time at day of the index child’s 10^th^ birthday and excluded all parents that had a cardiovascular outcome prior to beginning of the follow up period (n=263,057 mothers and n=27,854 fathers).

Random selections of children from multiple pregnancies were performed using PROC SURVEY SELECT in SAS 9.2 [8]. Distributions of parity were similar between CP and non-CP parents: first born (54% CP, 51% non-CP), second born (33% CP, 38% non-CP), and third or later child (13% CP, 11% non-CP).

### Congenital Cerebral Palsy

Children were identified as having *validated CP* if they were alive after the first year of life and were included in The Danish National Cerebral Palsy Registry. The Danish National Cerebral Palsy Registry is a population-based registry that contains records of individuals with CP from birth year 1925 and has reported birth prevalence of CP since 1950 [9]. Originally the database covered only patients from Eastern Denmark, about 50% of the Danish population, but since birth year 1995 the register became a national registry and was expanded to cover the entire newborn Danish population from then on. Medical histories of potential CP cases are collected from pediatric departments and cases of CP are validated by a child neurologist and an obstetrician based on review of the child’s physical findings recorded in medical records [9] . 

### Hospital Reported Diseases

The Danish National Hospital Register holds information on all discharges from Danish hospitals since 1977 and has included outpatient treatments since 1995[10]. All treatments in Danish hospitals are free of charge for residents. Diagnostic information, reported to the register after each hospital visit, is based on the Danish version of the *International Classification of Diseases*, 8^th^ Revision (ICD-8) and then 10^th^ Revision (ICD-10) from 1994 onward. 

Data on cardiovascular events, as diagnosed either as inpatient or as outpatient, were obtained from the Danish National Hospital Register. We examined all cardiovascular events together and performed separate analyses for hypertension, ischemic heart disease, cerebrovascular disease, and other cardiovascular disease (see Table S1 for ICD codes). Women could be counted in more than one cardiovascular subgroup, but for overall cardiovascular disease endpoints and within each subgroup, first diagnostic event was used. 

### Potential Confounders, Intermediate Factors, and Covariates

Information on child’s sex at birth (male/female), year of birth, gestational age, and birth weight was obtained from The Danish Medical Birth Registry. Information on gestational age at birth was estimated from last menstrual period, but ultrasound measures during early pregnancy have been used to estimate gestational age in most pregnancies in the last 20 years[11]. Preterm births in our study were those delivered before 37 weeks of gestation. Small for gestational age were identified as those weighing less than the 10th percentile for sex specific gestational age, based on the distribution available in the entire Danish Medical Birth Registry. We used the Danish Civil Registration System Registry to obtain information on place of child’s residence (east or west Denmark) at time of diagnosis of CP for CP children. For non-CP children and CP children without this information, residence at one year after birth (date of birth +365) was used, or otherwise first known residence was used. We coded east vs. west Denmark according to postal codes as a dichotomous variable and adjusted for it in our analyses. For parental education, we used information on each parent’s education at year of birth of the child or up to five years before and after. We then combined the parents’ educations, and used the higher education as a marker of socio-economic status (low, middle, and high). Fathers and mothers had equal education levels for 45% of the parents, 26% had fathers with a higher education level than mothers, and 29% had mothers with a higher education level than fathers; however in 4% of this latter group, fathers’ identification was not available. We used The Danish National Hospital Registry to ascertain if the mother had hypertensive disorder of pregnancy during the pregnancy of the child of interest (see Table S1 for ICD codes). 

Additional information about the registries is available at www.dst.dk. This study was approved by the Danish Data Protection Agency, Research Ethic Committee and University of California, Los Angeles Institutional Review Board.

### Statistical Analysis

Characteristics for maternal and paternal cohorts stratified by the sex of the child were analyzed as proportions, means ± standard deviations. We modeled the risk of cardiovascular diagnosis during the follow up time separately for mothers and fathers with a CP child compared to mothers and fathers who had a child without CP. All parents were followed from child’s 10^th^ birthday until a 1^st^ reported diagnosis of cardiovascular outcome, death, emigration or end of follow up (December 31, 2006), whichever occurred first. Hazard ratios and 95% confidence intervals (CI) were estimated by Cox proportional hazard regression models. For each cardiovascular subtype and all cause mortality, 1^st^ reported diagnosis of that outcome, death, emigration or end of follow up was used. 

In addition to unadjusted models, two models were examined with incremental multivariable adjustment for covariates:

1. Model 1: Models included calendar year at the start of follow up, child’s sex (male, female), child’s resident status in East Denmark (yes, no), parent’s age at start of follow up (year), parental education at year of birth of the child (low, middle, high),
2. Model 2-4: Models included all listed in Model 1 plus either maternal hypertensive disorder of pregnancy (yes, no), child small for gestational age (yes, no), or preterm birth (yes, no). Model 5 included all listed in Model 1 plus maternal hypertensive disorder of pregnancy (yes, no), child small for gestational age (yes, no) and preterm birth (yes, no).

Multiple imputation methods (“PROC MI and PROC MIANALYZE” in SAS 9.2[8]) were used to replace missing covariate data. The procedure generates 5 different simulated completed datasets, replacing each missing value with a set of plausible values based on the other available values for that variable and other covariate data. The multiply imputed data sets are then analyzed by using standard procedures for complete data and combining the results from these analyses. Missing values requiring imputation included: residence (n=12, <1%), parents’ education (n=99,057, 13%), gestational age (n=243,124, 33%), and small for gestational age (n=243,780, 33%). Plots of log [-log (survival rate)] against log (survival time) were used to check proportionality assumptions. All statistical analyses were conducted with SAS version 9.2[8].

Sensitivity analysis based on the methods of Arah et al[12] was performed for the fully adjusted model of maternal associations between having a male child with CP and a cerebrovascular event. We adjusted for smoking as an unknown confounder using priors derived from estimates of the association between smoking and CP in the Danish population, and between smoking and stroke from a similar Nordic population[13]. 

## Results

The construction of the cohort is shown in Figure 1. The cohorts of parents of CP children and non-CP children were identically selected with the exception of an addition of 8 CP children who died or emigrated during the follow up, but were kept in the analysis because information on the parents was available. We identified 733,730 mothers and 666,652 fathers of singletons that survived or did not emigrate prior to 10 years of age, among whom 1,592 and 1,484, respectively, had a child with CP. Parent descriptive data by sex of the child is shown in table 1. In our study, CP infants were more likely to be small for gestational age or to be born after a pregnancy complicated by preterm birth or hypertensive disorders of pregnancy. There was, as expected, a larger number of CP male children but no sex difference in the distribution of any of the factors studied. 

**Table 1:** Characteristics of the cohorts defined by sex of the parent and sex and diagnosis of CP in the child.

	**Maternal Cohort**				**Paternal Cohort**			
**Index child male**	**No**	**Yes**	**No**	**Yes**	**No**	**Yes**	**No**	**Yes**
**Index child with CP**	**No**	**No**	**Yes**	**Yes**	**No**	**No**	**Yes**	**Yes**
**N**	375,170	357,135	944	647	340,949	324,513	874	609
**Maternal Age (years**)*	37±5	37±5	37±5	37±5	40±6	40±6	40±6	40±6
**Education (%)**								
**Low**	22	22	27	29	21	21	25	28
**Medium**	46	46	45	42	46	47	46	42
**High**	32	32	28	29	33	32	29	30
**East Denmark Resident (%)**	52	52	91	92	51	51	90	92
**Preterm (%)**	4.8	4.1	37.8	39.8	4.7	4.0	36.7	39.4
**Small for Gestational Age (%)**	8.3	8.9	17.1	17.8	8.1	8.6	17.7	18.4
**Hypertensive Disorders of Pregnancy (%)**	4.1	4.0	6.9	6.2	4.1	4.0	7.1	6.1

* Mean + SD

For mothers, the average follow up time was 12±7 years for non-CP mothers and 10±7 for CP mothers with a maximum of 24 years of follow up time for both groups. At end of follow up, maternal age averaged at 50±8 years for non-CP mothers and 47±8 for CP mothers, with 27% and 17% of mothers, respectively, over age 55. At end of follow up, paternal age averaged at 52±9 years for non-CP, and 50±8 years for CP fathers with 36% of non-CP and 26% of CP fathers over age 55.

After adjustment for mother’s age, calendar year, child’s residence, parental education and child sex, the “all cardiovascular disease” endpoint was only significantly associated with CP in the cohort of mothers of male children (Table 2). The strength of the association was essentially unchanged after additional adjustment for small for gestational age offspring and hypertension disorder of pregnancy, but was attenuated after adjustment for preterm delivery (Table 3). 

**Table 2:** Hazard ratio (HR) for cardiovascular disease diagnosis in parents of CP children by gender of the parent and gender of the child.

		**Total**	**Cases**		**UnadjustedHR**		**Model 1**		
							**aHR**	**95%CI**	
**Mother of male child**									
**With non-CP child** (**ref**)		358,494+16,676	16,677						
**With CP child**		893+51	51		1.64		1.52	1.16 - 2.00	
**Mother of female child**									
**With non-CP child** (**ref**)		341,389+15,746	15,746						
**With CP child**		622+25	25		1.15		1.06	0.72 - 1.57	
**Father of male child**									
**With non-CP child** (**ref**)		311,328+29,621	29,621						
**With CP child**		813+61	61		1.07		1.03	0.80 - 1.32	
**Father of female child**									
**With non-CP child** (**ref**)		296,333+28,180	28,201						
**With CP child**		575+34	34		0.82		0.77	0.55 - 1.07	

Model 1: adjusted for at entry time: mother’s age, calendar year, child’s residence (east or west), parents’ education (low, middle, and high)

Reference group is parents of children with no CP per subgroup.

**Table 3:** Hazard ratio (HR) for cardiovascular disease diagnoses in mothers of a male CP child with different adjustment models.

Model	Factor for which factors is the model adjusted	aHR	95%CI
Model 1	At entry time: mother’s age, calendar year, child’s residence (east or west), parents’ education	1.52	1.16 -2.00
Model 2	Model 1 + hypertension disorders during pregnancy	1.52	1.15 - 1.99
Model 3	Model 1 + for small for gestational age	1.50	1.14 - 1.97
Model 4	Model 1 + for preterm delivery	1.34	1.02 - 1.76
Model 5	Model 1 + for hypertension disorders during pregnancy, small for gestational age and preterm delivery	1.34	1.02 - 1.76

Table 4 shows the associations of having a child with CP with different types of cardiovascular disease endpoints and all cause mortality in mothers of male children. After adjustment for mother’s age, calendar year, child’s residence, parental education and child sex, significant associations with CP were seen for hypertension and cerebrovascular disease. In the fully adjusted model, including adjustment for small for gestational age offspring, hypertension disorder of pregnancy and preterm delivery, the association with having a child with CP remained significant only for cerebrovascular disease. An analysis of the ICD codes for cerebrovascular disease in the mothers of male CP children showed seven ischemic strokes or transient ischemic attacks and two hemorrhagic strokes. In a fully adjusted analysis including adjustment for preterm delivery, having a male CP child showed a significant association with ischemic events. An analysis of risk factors for cerebrovascular event, diagnosed within five years prior to the event, was performed in mothers of male children having sustained a cerebrovascular event. Of the 1,301 women with cerebrovascular events and no children with CP, 77 had migraine, 17 had supraventricular arrhythmias including atrial fibrillation and 182 had hypertension. None of the 9 mothers of children with CP that had a cerebrovascular event had any of these diagnoses. However, in mothers that had a cerebrovascular event, diabetes mellitus was present in 175 mothers of non CP children (13.5 %) and 3 mothers of CP children (33 %). 

**Table 4:** Hazard ratio of specific cardiovascular disease diagnoses in mothers of a male CP child.

	Number of subjects studied				HR	Model 1		Model 5	
Cardiovascular Diagnosis	No	Yes	No	Yes		aHR	95%CI	aHR	95%CI
Index child with CP	No	No	Yes	Yes					
Hypertension	367,407	7,763	920	24	1.64	1.49	1.00 - 2.23	1.42	0.95 - 2.13
Ischemic Heart Disease	367,551	7,619	928	16	1.14	0.97	0.59 - 1.58	0.82	0.50 - 1.34
Cerebrovascular Disease	373,869	1,301	935	9	3.88	3.41	1.77 - 6.59	2.73	1.45 - 5.12
Ischemic Events	374,333	837	937	7	4.82	4.07	1.93 - 8.60	2.98	1.48 - 6.03
Other Cardiovascular Disorders	372,967	2,203	936	8	2.01	1.99	0.99 - 4.00	1.72	0.85 - 3.47
All Cause Mortality	363,752	11,418	919	25	1.16	1.10	0.74 - 1.63	0.94	0.64 - 1.40

Model 1: adjusted for at entry time: mother’s age, calendar year, child’s residence and parents’ education,

Model 5: adjusted for Model 1 + preterm delivery, hypertensive disorders during pregnancy and small for gestational age

Smoking data were not available to us. Since smoking more than 10 cigarettes a day is strongly associated with the risk of have an offspring with CP[14] and with an increased risk of cerebrovascular disease, a sensitivity analysis was conducted to estimate if confounding by smoking could explain the association. For estimating prior probabilities in our analysis, we used age adjusted risk ratios on the association between smoking and stroke in 45,449 Swedish women under 60 years of age (HR for 10+ cigarettes per day: 3.0, 95%CI: 1.9-4.6, as compared to non-smoking)[13]. We also used prevalence for exposure to smoking in CP and non-CP populations based on data from previous studies in a Danish cohort [4] showing that among non-CP parents, 13% smoked 10 or more cigarettes per day, while in CP parents, the percentage was 19%. Using this information along with Monte-Carlo sensitivity analysis, the full adjusted hazard ratio of cerebrovascular disease among women with a male child with CP was attenuated but remained significant, from aHR:1.41 (95%CI: 2.76-5.39) to aHR: 1.30 (95%CI: 2.66-5.43). These results suggest that within a range of plausible assumptions about the correlation between smoking and CP or cerebrovascular disease, it is unlikely that the associations we present between CP and maternal cerebrovascular disease are entirely explained by confounding by smoking. 

## Discussion

In this large contemporary Danish birth cohort, we observed an increased risk of cardiovascular disease in mothers who had male children with CP. The association was mostly attributable to an increased risk of hypertension and cerebrovascular disease. The risk of ischemic cerebrovascular events increased three fold in mothers of sons with CP and was only minimally attenuated by adjustment for small for gestational age offspring, hypertension disorder of pregnancy and preterm delivery. Moreover, traditional risk factors for cerebrovascular disease, present in mothers of non-CP sons, were not present in mothers of CP sons, suggesting that cerebrovascular disease in mothers of children with CP might have a different pathogenesis.

Lower parents’ socioeconomic status [15] and higher maternal age[4,16] have all been reported to increase risk of CP. Lower socioeconomic status and higher age are also known risk factors for cardiovascular disease. Our data show a lower percent of highly educated parents among children with CP. However, the association between having a child with CP and cardiovascular disease in mothers remained significant after adjustment for education level and parental age. 

Hypertensive disorders of pregnancy are associated with increased risk of having a CP child in term pregnancies[17] or when data are analyzed without stratification by gestational age[4]. It is also known that mothers with pregnancy hypertensive disorders are more likely to have or get cardiovascular risk factors[18-21] or diabetes[22] and have a markedly increased risk of stroke [23,24], ischemic heart disease[24,25] and cardiovascular death[26]. Similarly, children born small for gestational age are at increased risk of CP[15,27,28] and their parents have a higher risk of cardiovascular disease[29-31]. None of these associations have been reported to be modified by the sex of the child. After adjustment for potential confounders including hypertensive disorders of pregnancy, and small for gestational age, CP and cardiovascular diseases remained significantly associated in mothers of male children.

Preterm birth was noted to be associated with a higher risk of CP by the first authors describing the disease[32]. Using part of the same cohort as in our study, Catov et al found that women with a prior preterm birth had excess cardiovascular disease after adjustment for age, parity, and education[33]. In another study, Lykke et al also showed that mothers with preterm deliveries have twice the risk of cardiovascular death in long-term follow up studies[34]. After adjustment for preterm delivery in our study, the association between cardiovascular disease and CP was attenuated. This is expected if the association is related to preterm delivery, if preterm delivery is a strong confounder or if unknown confounders (e.g. genetic factors) are associated with both preterm delivery and/or cardiovascular disease. When studying if genetic factors have a direct effect on CP and cardiovascular disease, preterm should not be adjusted for. Preterm birth may be an intermediate between the gene and CP. If included in the model, adjusting for preterm birth creates the possibility of collider bias by linking both the genetic factor and other causes of preterm birth (like infections) that could cause CP.

Our data show that hypertension and cerebrovascular disease (particularly ischemic events) had significantly higher incidence in mothers of male children if the child had CP. The association between CP and cerebrovascular events continued to be statistically significant after adjustment for all available confounders. The increased risk of cerebrovascular disease in young mothers of a male child with CP, irrespective of gestational age at delivery requires further confirmation and analysis. The genetic or acquired proinflammatory or prothrombotic factor that potentially could account for these associations should be considered, but we should take into consideration that most of the parents had not yet reached the age of high risk of cardiovascular disease. Genetic causes have been proposed as a likely important etiologic factor of CP[35]. Prothrombotic or proinflammatory candidate genes for CP such as TGF-Beta-1[36], MMP-2[37], MTFHR-C677T[38], and TNF-alpha 308[39], have all been associated with increased cerebrovascular disease risk or worse cerebrovascular disease prognosis. Recently, an increased risk for neurodevelopmental disorders was reported in the offspring of mothers with antiphospholipid syndrome[40], a prothrombotic disorder strongly associated with cerebrovascular disease in young women[41].

Environmental factors could have contributed to the associations presented between cardiovascular diseases in the parents and CP in the offspring. Maternal stress or other risk factors associated with the care of a child with CP may act as an intermediate and lead to an increased risk of cardiovascular risk factors and cardiovascular disease. Factors such as stress[42] or changing dietary or exercise habits may be considered, but such associations are expected to be present equally in both boys or girls. Caregivers of children with CP, as compared with parents of children without disabilities, have been documented to have a very high “parental stress index”[43], or high salivary cortisol as an objective index of marked stress[44]. There is also a greater likelihood of a variety of physical problems, including back problems, migraine headaches, peptic ulcer, asthma and rheumatic disorders[45] in these caregivers. Prenatal maternal stress may also act as a confounder in the association between having a child with CP and cardiovascular outcome, as stress is a known risk factor for cardiovascular disease and Li et al also found that maternal exposure to bereavement after the loss of a child during the prenatal period was associated with increased risk of CP in children born either preterm or intrauterine growth restricted[46]. Moreover, the same group found that this type of stress results in an increased risk of attention deficit and hyperactivity disorders in male but not in female children[47]. Similar data were reported by Khasan et al for affective disorders in male offspring[48]. Other environmental factors, such as smoking can act as a confounder or provide a gene environment interaction in the association between CP and maternal risk of cardiovascular disease. The result of our sensitivity analysis, however, indicates that it is less likely that this hypothesis completely explains our results, but our simulated data on smoking cannot replace actual data and hence we do not know the true effect of this potential confounder. 

Possible explanations for the association between CP and cardiovascular disease occurring only in mothers of male children are: 1) the presence of an X-linked recessive gene associated with both cardiovascular disease and CP, 2) much stronger other risk factors for cardiovascular disease in fathers possibly explaining the lack of a statistically significant association between having a child with CP and cardiovascular disease, 3) an interaction between genes associated with cardiovascular disease and unknown factors determining the increased prevalence of male children among CP cases, or 4) maternal stress due to having a male child with CP increasing risk of cardiovascular event, as discussed above. Although there have been no reports concerning this latter type of gender disparity in CP, studies in other developmental disorders have reported an association between prenatal exposures and child sex. Nutritional deficiency has been reported to increase the risk of spinal bifida deaths[49] and late life depression[50] in male but not female offspring. Schizophrenia occurs predominantly in male children when the mother has autoimmune disorders[51] or when RhD incompatibility is present[52,53]. Moreover, the mothers’ immune response to the fetus during critical periods of development may have effects on the adult brain in a sex specific manner [54]. 

This is the first study addressing the cardiovascular risk in parents of children with CP. The main strength of the study was that it used the Danish National Cerebral Palsy Register to ascertain exposure to having a child with CP, which would make misclassification of the exposure less likely. The study has, however, limitations due to the construction of the cohort, due to the availability of cardiovascular diagnoses and due to availability of confounder data.

The cohort included a relatively small number of CP children and the follow up time was relatively short. Consequently most of the mothers and fathers had not reached age of the highest risk of cardiovascular disease outcomes and the study is limited to small number of cases occurring in relatively young subjects. In order to maximize the number of CP parents, we included parents of CP children who died or emigrated prior to their 10^th^ birthday, but we did not include parents of non-CP children of the same group. Also, prior to 1995, the CP registry did not cover Western Denmark and we tried to account for this by adjusting for residence of the child in the analysis. 

We used The Danish National Hospital Registry for data about cardiovascular events. Although underreporting of cardiovascular outcomes is less likely to be biased by socioeconomic status due to medical care being free of charge for patients in Denmark, some cardiovascular events such as hypertension did not require hospitalization and were therefore not recorded.

Our study is also limited by the fact that a number of confounders or intermediates such as smoking, body mass index, and gestational diabetes could not be accounted for. We used multiple imputation methods to fill in missing values in order to maximize the number of observations used in each analysis. Information on gestational age and education was missing for 33% and 13% of the children in our cohort respectively, and therefore imputation may have led to some loss of precision in the estimates and bias to the null. Additionally, although we used education as a marker of socio-economic status, there may still be residual confounding by socio-economic status that is not represented with this variable. Other unknown confounders may also be present and contribute to residual confounding.

In summary we have shown that having a male child with CP is associated with an increased risk of cardiovascular hospital diagnosed outcomes in the mother occurring at a relatively young age. Additional studies should be replicated in an independent data source as well as address exposure to genetic polymorphisms and the presence of cardiovascular risk factors in mothers of children with CP, which are necessary to explain the pathophysiology of this association.

## Supporting Information

Table S1 **Table of Diagnostic codes.**
(DOC)
