# Supplementary material for: Congenital Cerebral Palsy, Child Sex and Parent Cardiovascular Risk
Source: PLoS One. 2013 Nov 1;8(11):e79071. doi: 10.1371/journal.pone.0079071 (PMC3815096; doi:10.1371/journal.pone.0079071)
Supplement: Table S1 — Table of Diagnostic codes. (DOC) [file pone.0079071.s001.doc]

| **Table S1** | | |
| --- | --- | --- |
| **All Cardiovascular Disease** | icd-8 | icd-10 |
| **Atherosclerosis** | 44009: 44299, 44319, 44389:44599 | I70- I74, I79 |
| **Cerebrovascular Disease** | 43000: 43899 | I60-I69, G45-46 |
| **Ischemic Stroke** | 43200:43599, 43708:43899 | I63-I66, G45-46 |
| **Hypertension** | 40009: 40499 | I10, I12-I15 |
| **Ischemic Heart Disease** | 41009: 41499 | I20- I25 |
| **Other Cardiovascular** | 39099: 39899, 42000: 42499, 42600: 42609, 42720: 42797, 42908: 42909,  45099:45199  42599, 42709:42719, 42799:42900 | I0, I27-I41, I44-I49, I51-I52,  I26, I80  I11, I42-I43, I50 |
| **Supraventricular Arrhythmias** | 42720:42795 | I44-I48 |
| **Hypertensive Disorder During Pregnancy** | 63700, 63702- 63704, 63709, 63719 | O11, O13- O15 |
| **Diabetes Mellitus** | 24900- 25099 | E10-E14 |
| **Migraine** | 34600- 34699 | G43 |
